# Safety and feasibility of a layered embolization strategy with drug-eluting and uniform-caliber microspheres for advanced hepatocellular carcinoma and unresectable liver metastases: a prospective single-arm clinical trial

**DOI:** 10.3389/fonc.2026.1773259

**Published:** 2026-03-11

**Authors:** Xiangrui Chen, Louzong Sun, Min Hu, Ju Zhou, Chengluo Hao, Yunwei Han

**Affiliations:** 1 Department of Oncology, Zigong Third People’s Hospital, Zigong, Sichuan, China; 2 Department of Hepatobiliary Surgery, Zigong First People’s Hospital, Zigong, Sichuan, China; 3 Department of Dermatology, Zigong Third People’s Hospital, Zigong, Sichuan, China; 4 Department of Gastroenterology, Zigong Third People’s Hospital, Zigong, Sichuan, China; 5 Department of Oncology, Affiliated Traditional Chinese Medicine Hospital of Southwest Medical University, Luzhou, Sichuan, China

**Keywords:** drug-eluting microspheres, hepatocellular carcinoma, liver metastases, transarterial chemoembolization, uniform-caliber microspheres

## Abstract

**Background:**

Advanced hepatocellular carcinoma (HCC) and unresectable liver metastases present significant therapeutic challenges, particularly in patients with portal vein tumor thrombus or complex vascular architecture. This study evaluates the safety and feasibility of a novel layered embolization strategy combining drug-eluting and uniform-caliber microspheres.

**Methods:**

We conducted a prospective, single-arm clinical trial enrolling 33 patients with advanced HCC (BCLC stage C) or unresectable liver metastases and preserved liver function (Child-Pugh class A/B ≤7). The layered strategy involved initial embolization with 100-300 μm CalliSpheres^®^ drug-eluting microspheres loaded with epirubicin, followed by proximal flow blockade with 100/500/700 μm uniform-caliber blank microspheres. Primary endpoints were safety profiles and procedural feasibility; secondary endpoints included short-term imaging response and biomarker dynamics.

**Results:**

The procedure was technically successful in all patients. Treatment-related adverse events occurred in 28 patients (84.8%), with 96.4% being grade 1–2 according to CTCAE v5.0 criteria. The most common complications were hepatic function abnormalities (72.7%) and post-embolization syndrome (60.6%). All adverse events resolved with standard management within a median of 7 days. Among 27 evaluable patients, the disease control rate was 92.6% (7 partial responses, 18 stable disease, 2 progression). Tumor biomarker dynamics showed correlation with imaging response.

**Conclusions:**

This layered embolization strategy demonstrates an acceptable safety profile and technical feasibility in carefully selected patients with advanced liver tumors and preserved hepatic function. The findings provide a foundation for future randomized controlled trials to evaluate survival benefits and identify optimal patient subgroups.

## 1 Introduction

Hepatocellular carcinoma (HCC) remains the third leading cause of cancer-related mortality worldwide (1), with its incidence rising globally (2–4). Over 80% of cases are diagnosed at intermediate or advanced stages, where curative treatment options are limited. In China, chronic hepatitis B virus infection imposes a substantial disease burden, and transarterial chemoembolization (TACE) has evolved as a cornerstone locoregional therapy for unresectable HCC (5, 6), particularly for patients within Barcelona Clinic Liver Cancer (BCLC) stage B. However, for BCLC stage C HCC—characterized by vascular invasion, extrahepatic spread, or compromised performance status—the application of TACE remains controversial due to concerns regarding its safety and limited survival benefits (7). Concurrently, hepatic metastases from extrahepatic primary malignancies further complicate management, with limited effective locoregional therapeutic options available for liver-dominant metastatic disease.

Conventional TACE utilizing lipiodol-based chemotherapeutic emulsions often leads to significant post-embolization syndrome (PES) and uncontrolled drug release, resulting in suboptimal tumor control and hepatotoxicity. The advent of drug-eluting beads (DEB-TACE) improved pharmacokinetics through sustained intratumoral drug release (8–10), yet challenges persist in balancing complete tumor devascularization with preservation of functional hepatic parenchyma. Innovations in embolic materials, particularly uniform-caliber microspheres, enable size-based precise occlusion of tumor-feeding vessels, enhancing ischemic efficacy while minimizing non-target embolization. However, monotherapy using either drug-eluting microspheres or uniform-caliber microspheres alone may fail to address the heterogeneous vascular architecture of advanced tumors, especially in complex scenarios involving portal vein tumor thrombus (PVTT) or arteriovenous shunting.

An innovative “layered embolization” strategy has been proposed: initial deep intratumoral penetration using small-sized drug-eluting microspheres, followed by proximal flow blockade using larger uniform-caliber blank microspheres. This approach aims to maximize cytotoxic drug delivery while achieving complete hemodynamic occlusion, thereby improving the therapeutic index. Given the absence of clinical reports evaluating uniform-caliber blank microspheres in embolization therapy, this study aims to prospectively validate the safety and feasibility of this strategy for the first time.

Against this background, we conducted a prospective, single-arm clinical trial to systematically evaluate, for the first time, the application of a layered embolization protocol using CalliSpheres^®^ drug-eluting microspheres (100–300 μm) combined with uniform-caliber blank microspheres (100/500/700 μm) in patients with advanced primary HCC and unresectable hepatic metastases. This work addresses three core objectives: (1) safety profile in BCLC stage C patients and high-risk subgroups with anatomical variants such as PVTT; (2) correlation between early postoperative dynamics of hematological/inflammatory markers and tumor biomarkers (e.g., alpha-fetoprotein [AFP], protein induced by vitamin K absence or antagonist-II [PIVKA-II]) with short-term imaging response; (3) establishment of evidence-based standardized pathways for complication management. By rigorously selecting real-world patients with well-preserved hepatic functional reserve—a population frequently excluded from traditional registry trials—this study aims to provide the first systematic assessment of the acute safety profile and procedural feasibility of this layered embolization strategy, offering preliminary clinical evidence to refine TACE technical standards.

## 2 Methods

### 2.1 Study design​

This prospective, single-arm clinical study was conducted at the Department of Oncology, The Third People’s Hospital of Zigong City, Sichuan Province, China, from December 2024 to October 2025. The protocol was rigorously reviewed and approved by the Institutional Medical Ethics Committee (Approval No. IEC-AF/SW[Research]-07-3.0). This study was funded by the “2024 Key Science and Technology Project of Zigong City (Zigong Integrated Traditional Chinese and Western Medicine Research Institute Collaborative Innovation Category)” (Grant No.: 2024-ZXY-03-05). All procedures adhered to the principles of the Declaration of Helsinki and Chinese Good Clinical Practice guidelines.

**Figure 1:**
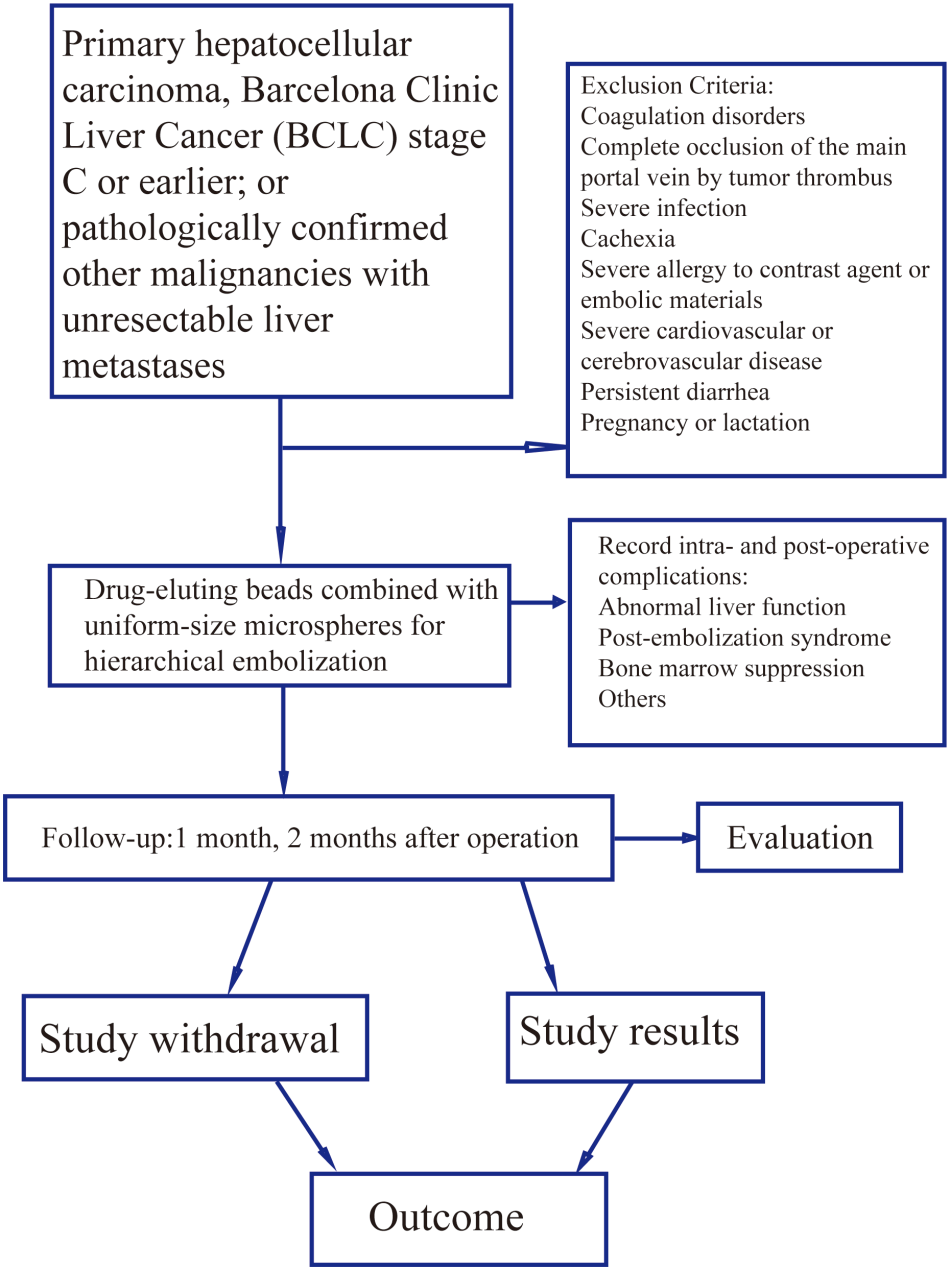
Flowchart of the study on hierarchical embolization with drug-eluting beads combined with uniform-size microspheres for the treatment of liver tumors. This is a prospective, single-arm exploratory study. The flowchart illustrates the entire process from patient screening, through treatment with hierarchical drug-eluting bead transarterial chemoembolization (DEB-TACE) using drug-eluting beads (loaded with chemotherapeutic agents) combined with uniform-size microspheres, to postoperative follow-up and evaluation. The main inclusion criteria consist of patients with pathologically or clinically confirmed unresectable primary hepatocellular carcinoma (BCLC stage C) or specified liver metastases, along with good liver function (Child-Pugh class A/B). Exclusion criteria are listed in the right section of the figure. All patients underwent scheduled follow-up at 1 and 2 months postoperatively to monitor complications and evaluate treatment efficacy.

This study aimed to evaluate the safety of TACE utilizing a layered embolization strategy with drug-eluting microspheres combined with uniform-caliber microspheres. As a first-in-human exploratory trial, its primary objective was to assess the safety profile of this strategy. The sample size was set at 33 patients based on the ICH E1 guidelines for the initial clinical evaluation of novel interventional techniques. This provides 80% power (α = 0.05) to detect treatment-related grade 3–4 adverse events with an incidence ≥10%, aligning with ethical requirements for first-in-human studies. The calculation was based on historical incidences of severe liver injury (8-15%) in prior TACE research, using a one-sided test to ensure identification of clinically significant safety signals. Specifically, with n=33, the study achieves 97.2% probability of observing ≥1 grade 3–4 AE if the true incidence is 10% (calculated as 1−(1−0.10)³³), exceeding the 80% power requirement. This aligns with ICH E1 recommendations for exploratory safety studies where precise incidence estimation is secondary to signal detection. An independent Data Safety Monitoring Board was established to periodically review safety data and had the authority to recommend early study termination based on the assessments. All patients provided written informed consent before enrollment, confirming their understanding of the treatment, potential risks, alternative options, and the right to withdraw at any time without affecting future care. The study flowchart is presented in **Figure 1**.

### 2.2 Patient selection

Inclusion criteria were: (1) age 18–80 years; (2) histologically confirmed or clinically diagnosed primary HCC with BCLC stage C or earlier, or pathologically confirmed unresectable hepatic metastases from other primary malignancies; (3) Eastern Cooperative Oncology Group (ECOG) performance status 0–2; (4) Child-Pugh class A or B (≤7 points); (5) expected survival >12 weeks; (6) adequate organ function: white blood cell count ≥3.0×10^9^/L, platelet count ≥50.0×10^9^/L, serum creatinine ≤176.8 μmol/L; (7) ability to complete follow-up. Although this cohort included both primary HCC and hepatic metastases, the primary objective was safety assessment of the embolization technique itself rather than tumor type-specific efficacy. Both entities share similar technical requirements for transarterial embolization in liver-dominant disease.

Exclusion criteria included: uncorrectable coagulopathy (international normalized ratio [INR] >1.5 or activated partial thromboplastin time [APTT] >1.5× upper limit of normal [ULN]); complete main portal vein occlusion by tumor thrombus with insufficient collateral circulation; concurrent active viral hepatitis (hepatitis B virus [HBV]-DNA >10³ IU/mL or hepatitis C virus [HCV]-RNA positive) or severe untreated infection; cachexia (body mass index [BMI] <18.5 kg/m²) or multi-organ failure; known severe allergy to chemotherapeutic agents, contrast media, or embolic materials used; unstable severe cardiovascular or cerebrovascular disease; persistent diarrhea (≥grade 1 lasting ≥14 days); pregnancy or lactation; and any condition deemed unsuitable by the investigator.

### 2.3 Layered embolization procedure

Preoperative preparation: All patients underwent contrast-enhanced liver computed tomography (CT) or magnetic resonance imaging (MRI) 1–3 days pre-procedure to delineate tumor location, size, number, vascular supply, and relationship to adjacent vessels. Patients fasted for 6–8 hours preoperatively. Intravenous access was established, and intravenous diazepam (5–10 mg) was administered preoperatively based on anxiety levels. Continuous monitoring of electrocardiogram, blood pressure, and oxygen saturation was maintained.

**Figure 2:**
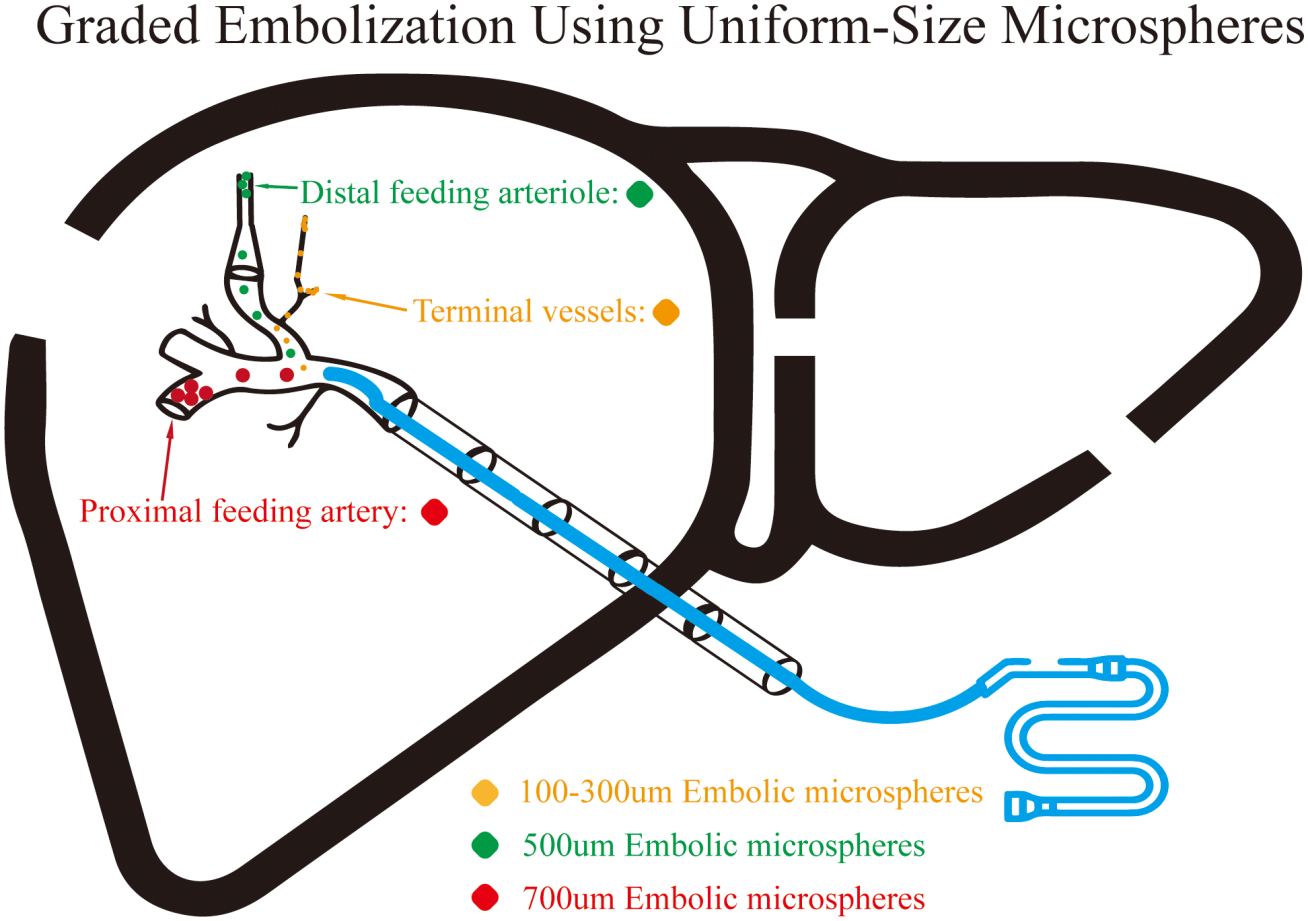
Schematic diagram of the layered embolization strategy using drug-eluting and uniform−size microspheres. This schematic visually illustrates the technical principle of the layered embolization strategy employing drug-eluting microspheres in combination with uniform−size microspheres. The embolization follows a “distal−to−proximal” approach. Initially, 100−300 μm drug−eluting microspheres (yellow dots) and 100 μm blank microspheres (supplement) are used to embolize the most distal tumor vasculature, achieving both local sustained chemotherapy release and terminal vessel occlusion. Subsequently, 500 μm (green dots) and 700 μm (red dots) uniform−size microspheres are sequentially administered to embolize the tumor−feeding arterioles and the proximal feeding arteries, respectively, to achieve complete blood flow blockade.

Procedure: Right femoral artery puncture was performed using modified Seldinger technique under local anesthesia, followed by 5F sheath insertion. Selective celiac trunk catheterization with a 5F-RH catheter was performed for digital subtraction angiography (DSA; Philips FD 2.0, Netherlands) to visualize tumor location, vascularity, and detect arteriovenous fistulae. Based on angiographic findings, superselective catheterization of tumor-feeding arteries was achieved using a 2.2F or 2.6F microcatheter system (Hengrui Galison, China). Chemotherapy infusion was administered first (HCC patients: modified FOLFOX regimen—folinic acid 500 mg + fluorouracil 500 mg + oxaliplatin 50 mg; hepatic metastases: regimen tailored to primary tumor, e.g., GP regimen—gemcitabine 800 mg + cisplatin 50 mg, or nab-paclitaxel 200 mg). Layer 1 embolization: 2 mL of 100–300 μm CalliSpheres^®^ drug-eluting microspheres (Hengrui Galison, China) pre-loaded with epirubicin (50 mg) was mixed with an equal volume of non-ionic contrast medium (iodixanol 320 mgI/mL) to form a suspension, infused slowly at 1–2 mL/min until significant reduction in tumor staining or blood flow deceleration. Layer 2 embolization: Sequential embolization using 100/500/700 μm uniform-caliber blank microspheres (Hengrui Hongyuan, China), diluted appropriately with contrast medium and injected slowly until significant flow reduction or stasis in the target vessel. Microsphere dosage was not pre-specified by fixed volume but determined intraoperatively based on real-time angiographic endpoints: cessation of tumor staining, significant flow deceleration, or microcatheter reflux—consistent with 2023 Chinese TACE clinical practice guidelines. Hepatic arteriography was repeated 5 minutes post-embolization to confirm completeness and exclude reflux or non-target embolization. A schematic illustration of the layered strategy is shown in **Figure 2**. The sheath was removed, and manual compression was applied to the puncture site for 6 hours.

Postoperative management: Patients remained supine for 24 hours with immobilization of the puncture-side limb for 6 hours. Intravenous hydration (2500–3000 mL/24h) was administered routinely. Hepatoprotective and antiemetic therapies were given based on embolization extent. Vital signs and abdominal symptoms were closely monitored, with analgesics administered via a stepwise protocol. Complete blood count and liver/kidney function tests were performed within 48 hours to assess early complications.

### 2.4 Follow-up plan and evaluation metrics

Follow-up visits occurred at 1 month (± 5 days) and 2 months post-procedure (primary safety endpoint). As an early safety assessment trial, we focused on acute safety events within 2 months post-treatment, consistent with evaluation standards for novel interventional strategies in radiology (Society of Interventional Radiology standards). Long-term survival and late complications will be assessed in subsequent studies. Each follow-up included: clinical symptom assessment; laboratory tests (complete blood count, liver function [albumin (ALB), total bilirubin (TBIL), alanine aminotransferase (ALT), aspartate aminotransferase (AST)], renal function [creatinine (Cr), blood urea nitrogen (BUN)], coagulation profile, tumor biomarkers [AFP, PIVKA-II, carcinoembryonic antigen (CEA), etc.]); and imaging (abdominal contrast-enhanced CT or MRI). Early imaging was permitted for symptomatic deterioration or suspected progression.

Primary efficacy endpoints were objective response rate (ORR) and disease control rate (DCR), assessed by modified Response Evaluation Criteria in Solid Tumors (mRECIST): complete response (CR) defined as disappearance of arterial enhancement in all target lesions; partial response (PR) as ≥30% decrease in the sum of diameters of target lesions with arterial enhancement; stable disease (SD) as neither PR nor progressive disease (PD) criteria met; PD as ≥20% increase in the sum of diameters or new lesions. Secondary efficacy endpoints included tumor biomarker response rate (≥50% decline from baseline defined as significant reduction) and symptom improvement (e.g., pain score, abdominal distension). For hepatic metastases, biomarkers were selected based on primary tumor type (e.g., CEA for colorectal cancer metastases). For exploratory biomarker analysis, we pre-specified the following endpoints based on EASL clinical practice guidelines: (1) biomarker response: ≥50% decline from baseline (clinically meaningful reduction associated with tumor necrosis); (2) biomarker progression: ≥100% increase (doubling, indicative of active tumor growth); (3) stable biomarker: change between -50% and +100%. These thresholds distinguish biologically relevant changes from assay variability (<20%).

### 2.5 Safety evaluation and complication management

Safety was graded using the National Cancer Institute Common Terminology Criteria for Adverse Events (CTCAE) version 5.0. All treatment-related AEs were recorded, with emphasis on hepatic function changes, PES (pain, fever, nausea/vomiting), and myelosuppression. Hepatic injury was defined as ALT/AST ≥3× ULN above baseline or new-onset jaundice. PES assessment included: pain (visual analog scale [VAS], 0–10); fever (temperature ≥37.5 °C); nausea/vomiting (frequency and severity). Myelosuppression was defined as white blood cell count <3.0×10^9^/L or platelet count <80×10^9^/L.

Standardized management protocols were established: Mild pain (VAS 1–3): oral non-steroidal anti-inflammatory drugs (celecoxib 200 mg twice daily). Moderate pain (VAS 4–6): weak opioids (tramadol 50–100 mg three times daily). Severe pain (VAS 7–10): strong opioids (morphine 5–10 mg every 6 hours). Fever management: physical cooling for temperature <38.5 °C; blood cultures and empirical antibiotics for temperature ≥38.5 °C or persistent fever >3 days. Nausea/vomiting: 5-HT3 receptor antagonist (ondansetron 8 mg intravenous), with intramuscular metoclopramide 10 mg added for persistent vomiting. Hepatic dysfunction: hepatoprotective agents; TACE deferred if ALT/AST >5× ULN until recovery. All serious adverse events (SAEs) were reported to the ethics committee within 24 hours, with treatment adjustment or termination if necessary. Special attention was paid to patients with PVTT or hepatic artery-portal vein fistula; these high-risk cases utilized meticulous superselective catheterization and reduced embolic doses.

### 2.6 Statistical analysis

Continuous variables were expressed as mean ± standard deviation and compared using paired t-tests, reporting t-values and two-sided p-values. Categorical variables were presented as counts (percentages) with descriptive analysis. All statistical tests were two-sided; p < 0.05 indicated statistical significance. Analyses were performed using SPSS 26.0 software. The primary focus was safety assessment of layered embolization with uniform-caliber microspheres. Paired t-tests analyzed postoperative hematological parameter changes (e.g., white blood cell count [WBC], C-reactive protein [CRP], ALT, AST). Descriptive statistics evaluated complication rates/severity and correlations between biomarker dynamics and imaging response. Safety data underwent systematic descriptive analysis, emphasizing complication types, incidence, severity, and management.

## 3 Results

### 3.1 Baseline characteristics of participants

As shown in **Table 1**, 33 patients with liver tumors underwent transarterial chemoembolization with uniform-caliber microspheres between December 2024 and October 2025. The cohort was predominantly male (81.8%) with a mean age of 63.7 ± 9.1 years. Most patients (81.8%) had primary hepatocellular carcinoma; 18.2% had hepatic metastases (primarily from lung squamous cell carcinoma). Most presented with advanced disease: 87.9% were T3–T4 stage, and 81.8% had distant metastases (M1 stage). According to the Barcelona Clinic Liver Cancer (BCLC) staging system—which integrates tumor burden, liver function, and performance status—all HCC patients were classified as stage C. The BCLC system was not applicable to patients with hepatic metastases from other primaries. Despite advanced staging, most maintained good performance status with stable vital signs (systolic blood pressure 132.9 ± 15.1 mmHg, diastolic blood pressure 82.4 ± 11.1 mmHg) and adequate baseline organ function.

**Table 1:** Baseline characteristics of patients undergoing transarterial chemoembolization with uniform caliber microspheres for liver tumors.

Variable	Value (Mean ± SD or n (%))
Age (years)​	63.7 ± 9.1
Systolic blood pressure (mmHg)​	132.9 ± 15.1
Diastolic blood pressure (mmHg)​	82.4 ± 11.1
White blood cell count (×10^9^/L)​	5.6 ± 2.1
Hemoglobin (g/L)​	119.8 ± 19.5
Platelet count (×10^9^/L)​	175.9 ± 84.8
Alanine aminotransferase (U/L)​	35.3 ± 24.3
Aspartate aminotransferase (U/L)​	56.2 ± 44.0
Albumin (g/L)​	38.1 ± 4.6
Total bilirubin (μmol/L)​	15.1 ± 9.2
Direct bilirubin (μmol/L)​	5.2 ± 3.7
Blood urea nitrogen (mmol/L)​	6.0 ± 1.6
Creatinine (μmol/L)​	67.7 ± 16.2
Uric acid (μmol/L)​	273.5 ± 79.2
C-reactive protein (mg/L)​	26.2 ± 35.5
Glucose (mmol/L)​	5.8 ± 1.2
Prothrombin time (s)​	12.5 ± 1.3
D-dimer (mg/L)​	1.8 ± 1.7
Tumor Marker	
PIVKA-II (mAU/mL)	5,683.2 ± 10,257.7
AFP (ng/mL)	592.7 ± 678.2
CEA (ng/mL)	8.3 ± 12.8
CA125 (U/mL)	67.2 ± 121.8
CA199 (U/mL)	40.5 ± 97.1
Gender​	
Male	27 (81.8%)
Female	6 (18.2%)
Child-Pugh Score​	
A (5–6 points)	32 (97.0%)
B (7–9 points)	1 (3.0%)
Tumor type	
Primary hepatocellular carcinoma	27 (81.8%)
Liver metastases	6 (18.2%)
BCLC staging	
C	27 (81.8%)
Not Applicable	6 (18.2%)
Treatment Compliance	
Planned ≥2 treatments	27 (81.8%)
Lost to follow-up after 1 treatment	6 (18.2%)

Baseline demographic, clinical, laboratory, and tumor characteristics of 33 patients with primary or metastatic liver tumors prior to treatment with uniform caliber microspheres. Data are presented as mean ± standard deviation or number (percentage). The n values in parentheses indicate the number of patients with available measurements for tumor markers. Child-Pugh classification was calculated based on bilirubin, albumin, and prothrombin time, with assumed absence of ascites and hepatic encephalopathy for all patients. PIVKA-II, protein induced by vitamin K absence or antagonist-II; AFP, alpha-fetoprotein; CEA, carcinoembryonic antigen; CA, cancer antigen.

Liver function was well-preserved: 97.0% were Child-Pugh class A (5–6 points), with only one patient (3.0%) in class B. Tumor biomarker profiles reflected underlying disease: 82.6% of tested patients had elevated PIVKA-II (mean 5,683.2 ± 10,257.7 mAU/mL), and 78.6% had elevated AFP (mean 592.7 ± 678.2 ng/mL). Hematological parameters showed mild anemia (hemoglobin 119.8 ± 19.5 g/L) with preserved platelet counts (175.9 ± 84.8 × 10^9^/L).

Treatment compliance was good: 81.8% planned ≥2 treatment sessions, while 18.2% were lost to follow-up after one treatment. Embolization predominantly used the FOLFOX regimen (folinic acid 500 mg + fluorouracil 500 mg + oxaliplatin 50 mg; 72.7%), followed by GP regimen (gemcitabine 800 mg + cisplatin 50 mg; 12.1%), nab-paclitaxel (200 mg; 9.1%), and others. This cohort represents a rigorously selected population with advanced liver tumors but preserved hepatic function, suitable for uniform-caliber microsphere TACE. Baseline characteristics reflect real-world practice where patients with adequate hepatic reserve are selected for locoregional therapy despite advanced tumor stages.

### 3.2 Postoperative changes in hematological parameters

**Table 2** summarizes short-term postoperative hematological changes. WBC and CRP increased significantly (ΔWBC: 5.18 ± 3.06 × 10^9^/L, t = 9.71, *p* < 0.001; ΔCRP: 29.12 ± 34.56 mg/L, t = 4.89, *p* < 0.001), indicating a procedure-induced inflammatory response. Hemoglobin decreased significantly (ΔHb: -4.45 ± 9.12 g/L, t = -2.84, *p =* 0.008), while platelet count changes were non-significant (ΔPLT: 4.79 ± 104.32 × 10^9^/L, t = 0.26, *p* = 0.796). Liver enzymes increased significantly (ΔALT: 72.61 ± 194.25 U/L, t = 2.15, *p* = 0.039; ΔAST: 118.48 ± 294.67 U/L, t = 2.35, *p* = 0.025), suggesting hepatocellular injury. ALB decreased significantly (ΔALB: -3.12 ± 3.45 g/L, t = -5.24, p < 0.001). Bilirubin markers also increased (ΔTBIL: 3.12 ± 8.76 μmol/L, t = 2.06, p = 0.048; ΔDBIL: 1.45 ± 3.21 μmol/L, t = 2.60, *p* = 0.014). Renal parameters (BUN, Cr, uric acid [UA]) showed no significant changes (all *p* > 0.05).

**Table 2:** Changes in hematological parameters after interventional procedure.

Parameter	Change value (Mean ± SD)	t-value	p-value
WBC (×10^9^/L)	5.18 ± 3.06	9.71	<0.001
HB (g/L)	-4.45 ± 9.12	-2.84	0.008
PLT (×10^9^/L)	4.79 ± 104.32	0.26	0.796
ALT (U/L)	72.61 ± 194.25	2.15	0.039
AST (U/L)	118.48 ± 294.67	2.35	0.025
ALB (g/L)	-3.12 ± 3.45	-5.24	<0.001
TBIL (μmol/L)	3.12 ± 8.76	2.06	0.048
DBIL (μmol/L)	1.45 ± 3.21	2.60	0.014
BUN (mmol/L)	0.56 ± 2.89	1.12	0.271
CR (μmol/L)	-0.85 ± 15.34	-0.32	0.754
UA (μmol/L)	-10.24 ± 50.12	-1.18	0.247
CRP (mg/L)	29.12 ± 34.56	4.89	<0.001

Values represent mean changes ± standard deviation, where change = postoperative value - preoperative value. P-values were derived from one-sample t-tests. WBC, white blood cell count; HB, hemoglobin; PLT, platelet count; ALT, alanine aminotransferase; AST, aspartate aminotransferase; ALB, albumin; TBIL, total bilirubin; DBIL, direct bilirubin; BUN, blood urea nitrogen; CR, creatinine; UA, uric acid; CRP, C-reactive protein. Statistical significance was defined as p < 0.05.

These findings indicate primary postoperative manifestations were inflammatory response (elevated WBC/CRP), hepatic injury (elevated ALT/AST), anemia (decreased Hb), and hypoalbuminemia (decreased ALB). Despite non-significant overall platelet change, substantial inter-individual variation necessitates personalized monitoring.

### 3.3 Analysis of perioperative complications

Primary safety endpoint: Among 33 treated patients, 28 (84.8%) experienced treatment-related AEs. Of these, 96.4% (27/28) were grade 1–2 (mild-to-moderate), and only one patient (3.0%) developed grade 3 AE (AST >5× ULN). No grade 4–5 AEs or treatment-related deaths occurred. All AEs resolved completely with standardized management, with a median recovery time of 7 days (range: 3–14 days). All procedures were completed successfully without intraoperative mortality or major complications (e.g., vascular injury, non-target embolization). Postoperative complication details are provided in **Table 3**.

**Table 3:** Summary and grading of treatment-related adverse events (n=33). .

Adverse events of TACE	Total cases, n	Grade 1, n	Grade 2, n	Grade 3, n	Grade 4-5, n
Any Treatment-Related Adverse Event	28	18	9	1	0
Hepatic Function Abnormalities	24	17	6	1	0
Elevated transaminases	23	16	6	1	0
Elevated bilirubin	1	1	0	0	0
Post-Embolization Syndrome​	20	17	3	0	0
Pain	13	11	2	0	0
Nausea/Vomiting	12	11	1	0	0
Fever	2	2	0	0	0
Myelosuppression	2	2	0	0	0
Other Adverse Events​	2	2	0	0	0
Constipation	2	2	0	0	0
Allergic reaction	0	0	0	0	0
Vascular injury	0	0	0	0	0
Vasovagal reaction	0	0	0	0	0
Non-target embolization	0	0	0	0	0
Lower extremity thrombosis	0	0	0	0	0
Puncture site hematoma	0	0	0	0	0
Paralysis	0	0	0	0	0
Cough	0	0	0	0	0
Dyspnea	0	0	0	0	0
Abscess formation	0	0	0	0	0
Renal impairment	0	0	0	0	0

Adverse events were graded according to the National Cancer Institute Common Terminology Criteria for Adverse Events (NCI CTCAE), version 5.0. “Post-Embolization Syndrome” encompasses fever, pain, and nausea/vomiting occurring within 72 hours post-procedure. Hepatic function abnormalities include elevated transaminases and/or bilirubin; subtypes are reported separately without overlap. Events with zero occurrences across all grades are listed for completeness but omitted from grading columns. Grade 4–5 denotes severe/life-threatening events or death related to treatment.

Hepatic function abnormalities occurred in 24 patients (72.7%), predominantly as elevated transaminases (23 patients, 69.7%) and elevated bilirubin (1 patient, 3.0%). Among transaminase elevations, 16 were grade 1 (48.5%), 6 were grade 2 (18.2%), and 1 was grade 3 (3.0%). Elevated bilirubin was grade 1 in the single case. Post-embolization syndrome (PES) was observed in 20 patients (60.6%), manifesting as pain (13 patients, 39.4%; grade1:11/33.3%, grade2:2/6.1%), nausea/vomiting (12 patients, 36.4%; grade1:11/33.3%, grade2:1/3.0%), and fever (2 patients, 6.1%; both grade1). Myelosuppression occurred in 2 patients (6.1%), both grade 1. Constipation was reported in 2 patients (6.1%), both grade 1. Critically, no cases of allergic reaction, vascular injury, vasovagal reaction, non-target embolization, lower extremity thrombosis, puncture site hematoma, paralysis, cough, dyspnea, abscess formation, or renal impairment were observed.

Further analysis showed transaminase elevations were transient, normalizing within 1–2 weeks with hepatoprotective therapy, suggesting controllable hepatic impact in selected patients. Pain typically occurred 2–6 hours post-procedure in the tumor-bearing region; 11 of 13 pain cases (mild-to-moderate) responded to non-steroidal anti-inflammatory drugs, while 2 moderate cases required weak opioids. Ten of 12 nausea/vomiting cases occurred after FOLFOX, implicating oxaliplatin in gastrointestinal toxicity. Both fever cases were low-grade (37.5–38.5 °C) without chills or persistent high fever, consistent with absorption fever from tumor necrosis, requiring no intervention.

In two high-risk patients with PVTT and hepatic artery-portal vein fistula, significant transaminase elevation occurred (reaching grade 2), but both normalized after one week of hepatoprotective therapy. Despite complex anatomy, no severe complications (e.g., non-target embolization, liver failure) developed, demonstrating controllable safety with thorough preoperative assessment and precise technique.

Overall, with strict patient selection, TACE complications primarily involve manageable PES and transient hepatic dysfunction, with severe events rare. The high proportion of Child-Pugh class A patients (97.0%) likely contributed to this favorable safety profile. Comprehensive preoperative assessment, individualized planning, and proactive perioperative management for high-risk patients effectively mitigate complications and improve tolerance.

### 3.4 Assessment of short-term efficacy after TACE

**Figure 3:**
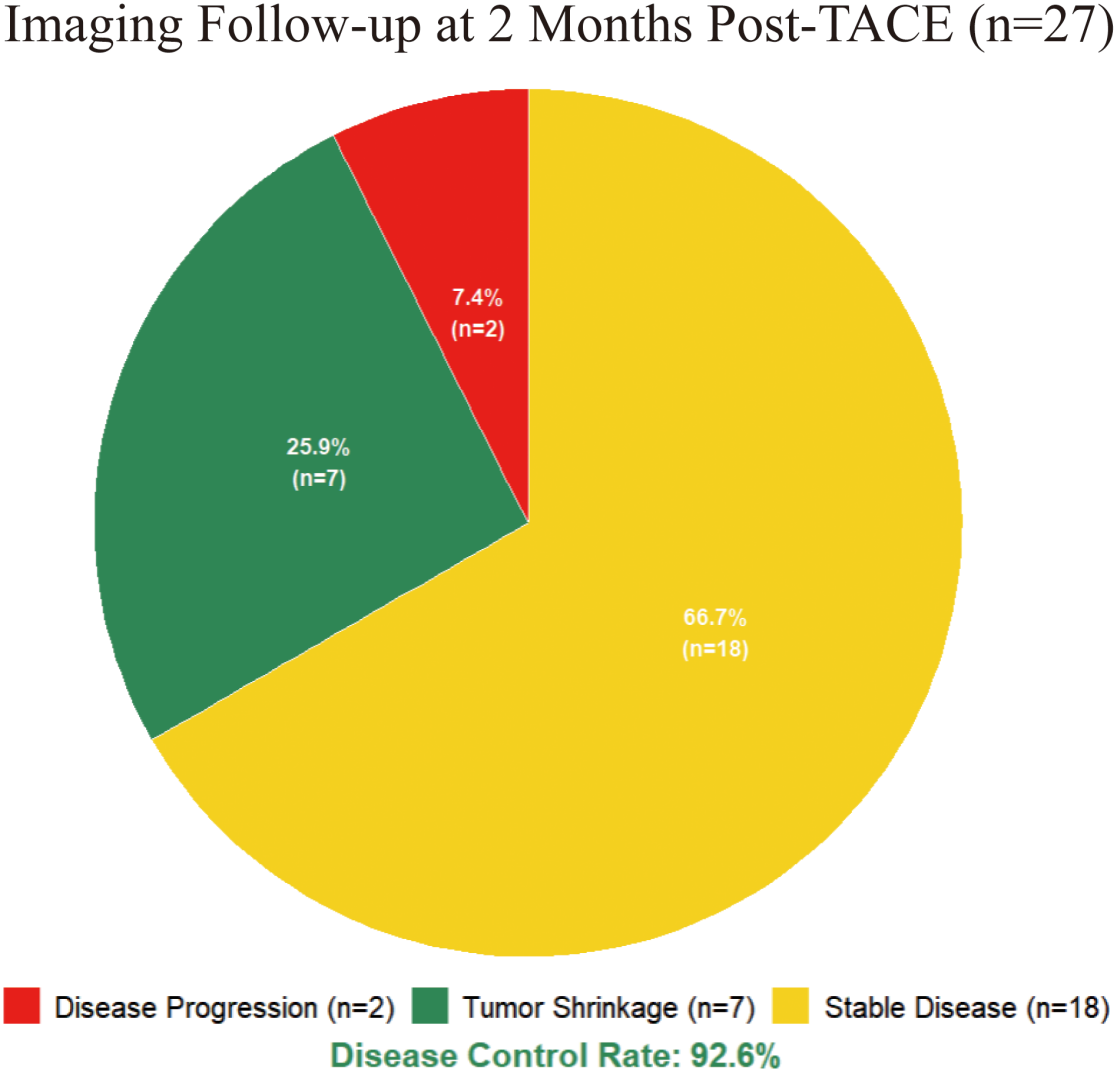
Imaging follow-up at 2 months post-TACE. This pie chart illustrates the radiological response assessment for the 27 patients who completed follow-up. The majority of patients (66.7%, n=18) achieved stable disease, while 25.9% (n=7) experienced Partial Response, resulting in a disease control rate of 92.6%. Disease progression was observed in 7.4% (n=2) of the evaluated cohort.

Twenty-seven patients (81.8%) completed imaging follow-up within two months post-procedure. Radiological assessment (**Figure 3**) showed Partial Response in 7 patients (25.9%), stable disease in 18 (66.7%), and progression in 2 (7.4%). Thus, ORR (shrinkage) was 25.9%, and DCR (shrinkage + stable) reached 92.6%. Six patients (18.2%) were lost to follow-up after initial treatment, lacking imaging data.

Exploratory analysis of the 22 HCC patients with evaluable biomarkers revealed a directional association between biomarker decline and imaging response (**Table 4**). Specifically, 57.1% (4/7) of PR patients achieved AFP response (≥50% decline) versus 21.4% (3/14) of SD patients; similarly, 75.0% (3/4) of PR patients with evaluable PIVKA-II achieved biomarker response versus 37.5% (3/8) of SD patients. Both progression cases exhibited stable or rising biomarkers: the HCC patient with PVTT and fistula showed no AFP/PIVKA-II decline despite imaging progression; the lung adenocarcinoma metastasis patient had CEA rise from 16.07 to 15.8 ng/mL. Spearman correlation analysis showed consistent directionality (rho=0.31 for AFP, rho=0.35 for PIVKA-II) though not statistically significant (p>0.05), supporting hypothesis generation for future validation studies.

**Table 4:** Association between tumor biomarker dynamics and imaging response in hepatocellular carcinoma patients (n=22).

Imaging response (mRECIST)	n	Change median (IQR)	Response rate*
AFP			
Partial response (PR)	7	-69.8% (-86.7 to -2.6)	4/7 (57.1%)
Stable disease (SD)	14	-20.4% (-48.1 to 0.0)	3/14 (21.4%)
Progressive disease (PD)	1	0.0%	0/1 (0%)
Spearman rho (p-value)	—	0.31 (0.158)	—
PIVKA-II			
Partial response (PR)	4	-63.6% (-84.3 to -20.4)	3/4 (75.0%)
Stable disease (SD)	8	-14.8% (-79.6 to +113.9)	3/8 (37.5%)
Progressive disease (PD)	1	0.0%	0/1 (0%)

*Biomarker response defined *a priori* as ≥50% decline from baseline. PIVKA-II evaluable in 13/22 HCC patients (59.1%) due to real-world clinical practice constraints (not routinely monitored per institutional protocol); 9 patients excluded due to missing baseline or follow-up values. Correlations did not reach statistical significance due to limited sample size, but directionality (greater biomarker decline in PR vs. SD vs. PD) supports hypothesis generation for future validation studies. This aligns with the exploratory nature of this first-in-human safety trial.

Notably, this single-arm safety trial was not designed to evaluate efficacy superiority or non-inferiority. Efficacy data are exploratory, intended to generate hypotheses for future studies.

## 4 Discussion

This study provides preliminary safety assessment of a layered embolization strategy combining drug-eluting and uniform-caliber blank microspheres in rigorously selected advanced liver tumor patients. The results indicate that the technique demonstrated good overall tolerability in patients with Child-Pugh class A liver function, with postoperative complications predominantly mild to moderate (96.4%) and mostly transient. Notably, hepatic function abnormalities were relatively common (72.7%), primarily manifesting as elevated transaminases (69.7%), but all cases returned to baseline levels following hepatoprotective therapy, suggesting the liver function impairment is reversible in populations with adequate hepatic reserve. This aligns with prior DEB-TACE safety reports (11–13), but extends evidence to high-risk anatomical variants like PVTT, suggesting cautious expansion of TACE indications.

Procedural details of layered embolization may influence safety (14). Two PVTT/fistula patients developed significant transaminase elevation (>120 U/L) but avoided liver failure, attributable to superselective catheterization and reduced embolic doses. The high gastrointestinal toxicity rate with FOLFOX (10/12 nausea/vomiting cases) underscores the need for regimen individualization. These observations align with prior TACE complication management guidelines (15–17), but this study is the first to systematically document procedural nuances and complication correlations in the context of uniform-caliber microspheres, providing empirical refinement guidance.

The layered technique—initial terminal embolization/drug release with 100–300 μm drug-eluting microspheres, followed by proximal reinforcement with 100/500/700 μm uniform-caliber microspheres—offers unique advantages. Uniform-caliber microspheres prevent proximal vessel occlusion by large particles (common in heterogeneous microspheres), enabling deeper penetration and more homogeneous tumor bed embolization. However, short-term efficacy requires cautious interpretation. Among 27 evaluable patients, DCR was 92.6%, but ORR was only 25.9%, consistent with historical TACE monotherapy data for HCC (ORR 20–35%) (18–20), indicating no significant efficacy breakthrough. Notably, biomarker dynamics correlated with imaging: 71.4% of patients with shrinkage showed ≥50% AFP/PIVKA-II decline, while progression cases had rising markers. However, limited sample size precludes establishing biomarkers as surrogate endpoints.

Key limitations exist: The single-arm design precludes direct comparison with conventional TACE. The small sample size (n = 33), particularly few hepatic metastases (n = 6), limits subgroup analysis. The 2-month follow-up is insufficient for long-term survival assessment. However, for a first-in-human interventional technique, this window adequately identifies acute safety signals and early feasibility. Additionally, 97.0% Child-Pugh class A patients limit generalizability to those with compromised liver function. These constraints mirror challenges in ethically conducting innovative interventional research. Furthermore, the inclusion of heterogeneous tumor histologies—though justified by the primary safety objective—limits generalizability across indications. Future trials should stratify by primary tumor type to identify histology-specific safety and efficacy profiles. This study did not perform formal health economic evaluation; future cost-effectiveness analyses comparing layered embolization versus conventional TACE are warranted to inform healthcare policy decisions.

In summary, for advanced liver tumor patients with preserved hepatic function, layered embolization with drug-eluting and uniform-caliber blank microspheres demonstrates an acceptable safety profile. Its procedural protocols and complication management may inform clinical practice. Future studies should validate survival benefits in larger cohorts and identify optimal patient subgroups. Current data suggest this technique may serve as a component of multidisciplinary management for Child-Pugh class A patients with branch PVTT, provided strict individualization principles are followed.

## 5 Conclusion

In rigorously selected advanced liver tumor patients with well-preserved liver function (97.0% Child-Pugh class A), the layered embolization strategy using drug-eluting and uniform-caliber blank microspheres demonstrated an acceptable acute safety profile: 84.8% experienced treatment-related adverse events, but 96.4% were grade 1–2, and all resolved completely with standardized management. Among 27 evaluable patients, a 92.6% disease control rate was observed; this exploratory efficacy signal requires validation in subsequent studies. This study provides the first systematic documentation of procedural details, complication spectrum, and standardized management pathways for this innovative strategy, offering crucial clinical reference for designing future multicenter randomized controlled trials.

## Data Availability

The original contributions presented in the study are included in the article/Supplementary Material. Further inquiries can be directed to the corresponding authors.
